# Students’ voices on spiritual care at a Higher Education Institution in the Western Cape

**DOI:** 10.4102/curationis.v38i2.1520

**Published:** 2015-12-18

**Authors:** Ntombizodwa S. Linda, Hester C. Klopper, Deliwe R. Phetlhu

**Affiliations:** 1 School of Nursing, University of the Western Cape, South Africa

## Abstract

**Background:**

Nurses have a moral obligation to ensure holistic care of patients, inclusive of the spiritual dimension. However, there seems to be a void in the teaching and learning of spiritual care in nursing curricula. Despite the South African Nursing Council being in favour of holistic nursing, there are no measures in place to ensure implementation of spiritual care, hence its practice is not standardised in nursing education in South Africa. Currently, the undergraduate nursing curriculum does not provide clear direction on how spiritual care in nursing should be integrated and the reason for this is not clear. It appears that the lack of professional regulation, difficulties in definition and the personalised nature of spiritual practice are partly responsible for the practice being barely enforced and scarcely practised by students in clinical placements. The aim of the study was to develop a practice theory for teaching–learning of spiritual care in the undergraduate nursing programme.

**Objectives:**

The study objective was to describe and explore the students’ experiencs of teaching–learning of spiritual care in the undergraduate nursing programme.

**Methods:**

A qualitative, explorative, descriptive and contextual design with purposive sampling was used. The sample consisted of undergraduate nursing students at a University in the Western Cape Province. Measures for trustworthiness were applied.

**Results:**

The findings indicated a need to provide support, a conducive learning environment and structure for teaching, learning and practice of spiritual care.

**Conclusion:**

There is a need for formal education regarding spiritual care in nursing.

## Introduction

Literature attests to the difficulty that exists with regard to the integration of spiritual care in undergraduate nursing curricula, pointing out various reasons why there is not a golden standard for spiritual care worldwide (Baldacchino & Draper 2001; Pesut 2002:128). Despite acknowledgements about the role and benefits of spiritual care in health and healing, challenges still exist with regard to how it should be taught and practised within the nursing profession (De La Porte 2014).

Spiritual needs can be understood as the invisible or inner aspect of humanity needs, including but not limited to meaning, hope, motivation, aspirations, purpose of life and meaningful experiences (MacKinlay & Trevitt 2007). If these needs are not nurtured they translate into negative emotions and deplete a person’s energy. Ill-health affects physiological functioning, which may further interfere with coping mechanisms that every person needs for daily survival. The lack of spiritually-focused care in nursing may prolong recovery from illness for certain patients because of mental stress, which affects physiological immunity and could be fatal. In addition, studies show that health professionals and care givers are not adequately and formally prepared on how to provide spiritual care; hence they lack confidence with regard to its provision (McSherry, Cash & Ross 2004; Taylor & Mamier 2005).

Lowry and Conco (2002:388), as well as Hsiao, Chiang and Chien (2010:386), argue that spiritual care becomes more important when a person is faced with life issues or life threatening circumstances that negatively challenge a person’s inner tranquility and peace, thus evoking spiritual discomfort or crisis in severe cases. If nurse practitioners, educators and researchers agree that patients may sometimes experience needs that cannot be quenched by mechanical interventions such as administration of pharmaceutical and surgical procedures, then nurses need to engage with the question of how nursing interventions can reach out to these inner needs.

It is evident from literature that there is a lack of formal education on spiritual health when preparing nurses for practice (Ross 1994; 2006; Van Leeuwen *et al*. 2006). This emanates from lack of curriculum clarity on spiritual care content in the nursing programmes. A lack of spiritual care training is not only a barrier in provision of spiritual care, but is equally responsible for nurses’ inability to detect patient’s spiritual needs (Narayanasamy 1999:275). Bloemhard (2008) reports that ignorant dismissal of spiritual care by nurses with no consideration of the value it has on ill-health and sickness, accounts for slow uptake of spiritual care practices. A similar argument is noted from different authors that, as a result of limited knowledge, nurses do not understand the importance of spiritual care and its value for a sick person (Van Leeuwen & Cusveller 2003:235).

Various challenges account for lack of education and training of spiritual care (Miner-Williams 2006:811), including the question of who qualifies to teach spiritual-related matters, as it is often assumed to be sensitive and a patient’s private territory. Adding to the complexity are nurse educators admitting to having difficulties in teaching and evaluation of the affective domain of the nursing knowledge. The affective domain in nursing is not only vital because it defines behaviours that correspond to attitude and values, but more importantly because it relates to attributes of cultivating values and ability to manage oneself. Further, some authors argue that an affect is an aspect of human nature which promotes awareness of important sensory and situational changes and motivates action. It is, therefore, a skill that is inseparable from nursing (Lopez & Snyder 2003). This is supported by Peterson and Seligman (2004) who agree that individuals who learn to recognise and engage their emotions are ready to grow affectively and can respond to challenges appropriately.

Efforts to instill affective skills in nurses were made through teaching of subjects like Ethics and Professional Practice as it was believed that they would influence spiritual and moral development of students and produce a nurse who could provide holistic nursing care (Monareng 2011:8). Nevertheless, discrepancy still exists between teaching of these subjects and the intended outcomes. Contrary to their expected outcomes, the content of these subjects has only been to alert the nurses about potential malpractice and unprofessional behaviour, respectively, but do not develop spiritual and moral competencies. Monareng (2011:14) argues that the education and training of nurses that do not consider the patient’s and student’s spirituality on such matters points to a serious gap in nursing education and practice.

This notion is supported by Tjale and Bruce (2007:46) who reject educational programmes that emphasise the systems approach at the expense of the spiritual and its influence on the wellbeing of the person. Clearly, nurse practitioners and nurse educators have not given spiritual care the attention it deserves in order to render nursing training and practice of a truly holistic nature (Tjale & Bruce 2007:46). This lack of education and training in spiritual care leads to the lack of implementation. This article acknowledges that the lack of adequate understanding of the role of spiritual care in nursing remains a serious challenge.

### Problem statement

Despite nursing education programmes being commended for keeping pace with the rapid advances in disease management, spiritual care has received far less attention in nursing curricula (Keefe 2005). This issue is evident by lack of clarity and direction on how the spiritual care in nursing should be taught and learned. The reason for this is not clear. Despite the South African Nursing Council (SANC) being in favour of holistic nursing, its implementation has not received much-needed attention in South Africa. As a result, teaching of spiritual care in the undergraduate programme is very seldom enforced and barely practised by students when they are in clinical placements. This oversight on teaching and learning of spiritual care in nursing causes students to lack foundational knowledge and required skills in this area of care. Tjale and Bruce (2007:46) argue that the lack of clear practice guidelines and directives from the SANC regarding spiritual aspects in the education and practice of nursing could be the cause of this oversight. The same challenge is blamed for the lack of practice of spiritual care worldwide (Carr 2008; Wallace *et al*. 2008:2).

#### Research objectives

One of the five objectives of the study that is addressed in this article was to describe and explore students’ opinions of teaching-learning of spiritual care in the undergraduate nursing programme.

## Research design

This study forms part of a larger theory-generative study where a qualitative, explorative, descriptive and contextual design was used. The qualitative approach enhanced collection of rich data from participants through thick description of participants’ voices and use of excerpts to support the study findings (Lincoln & Guba 1985).

Focus groups (FG) discussion, as described by Morgan (1998), Krueger (2002:7) and Litosseliti (2003) was used to collect data from undergraduate nursing students during the first phase of the research study.

### Population

The population for the study comprised the nursing students in the undergraduate nursing programme years 1 to 4 who were registered for a Bachelor’s Degree under the prescription of the regulation R425 by the SANC (1985) at a higher education institution in the Western Cape Province. The total population size was 1009 students.

### Sample and sampling method

A non-probability, purposive-convenient sampling method (Brink 2011:102) was used to form a stratified sample of 90 nursing students from the total population. The student participants were therefore dispersed in different sample clusters based on the year level of the education and training programme. The student nurses were included based on receipt of written informed consent and proof that they were registered for the Basic Nursing programme that was offered under R425 (SANC 1985) as regulated by SANC and doing nursing modules at the time of the interviews. Exclusion was based on failure to meet the inclusion criteria.

### Data treatment

Data were collected using FG interviews. Nine FG interviews were conducted with students from the first to final year of the nursing education and training programme. Saturation guided termination of data collection. Data were transcribed verbatim and transcript uploaded on AtlasTi7 (Foxit Corporation 2003–2012) to further manage the raw data. Memos, families’ anecdotes and quotes were developed and enhanced reduction of data and development themes. Data were further analysed and synthesised using Tesch’s (1992) reasoning strategy and inductive-deductive reasoning process (Creswell 2009). Tesch’s (1992) coding method allowed the researcher to arrange the categories, patterns and themes as they emerged for raw data.

## Results

This article presents only the findings from the first- to fourth-year undergraduate nursing students who participated in FG interviews. Most students reflected on their own lives about the concept of spirituality as a point of departure. It was obvious that the concept of spiritual care under discussion could not easily be embarked upon without them discussing spirituality in general. Because of the complex nature of the discussions that emerged, only the students’ experiences with regard to the teaching and learning of spiritual care will be presented in this article. Subthemes that emerged for teaching and learning of spiritual care are: (1) Personal inferences about learning spiritual care; (2) Perceived challenges in learning of spiritual care; (3) Perceived conflicting nursing perspectives; (4) Students’ spiritual and emotional needs.

### Personal inferences about learning spiritual care

Students reported that the subject of spiritual care was addressed mainly in their first year of the programme, but not in depth. Spiritual care learning content was perceived as pieces of information which related to loss and grieving and religious requirements of different religions. Although students confirmed that they learnt about death and dying and how to deal with the bereaved patient’s family, they felt it was not sufficient. The fourth-year students said:

‘But it was just like an introduction, we did not do much.’ (Male student, 25 years old, 4th year)

‘We [*students*] were just introduced to different types of religions but nothing further than that.’ (Female student, 27 years old, 4th year)

‘Spiritual care was done here and there it was not continuous …, it covered the subject of death and dying, bereaved family, as well as patient’s religious requirement or practices primarily.’ (Female student, 27 years old, 4th year)

Students acknowledged that the subject was addressed only briefly and it was generally perceived as introductory lessons which enabled them to gain some understanding and an experience of caring for a dying patient and bereaved family.

The lack of depth of the learning content of spiritual care is evidenced in students’ lack of purposeful intervention when taking care of a patient’s spiritual or religious needs. Student discussion demonstrated that they did not possess appropriate or personal knowledge as to why they were expected to provide religious and/or spiritual care to patients. This observation was made from students’ failure to comprehend the purpose and intent of interventions oriented toward religion, beyond attending to religious requirements. For example, they did not have knowledge as to what would happen to a Muslim patient if he or she was given pork by error or was not allowed to go for prayer. They said they had never reflected on that before. These were simple notions that depicted students as not possessing sufficient understanding as to why they had to attend to a patient`s religious needs in the first place. Final-year students said they were unable to recall the content of what they learned on the subject of spiritual care. According to them, the programme is more focused on professionalism. Such an observation for a final-year student is appropriate with regard to their level of training, where professionalism and ward administration are their learning focus. However, the fact that they could not remember what, when and how they learned with regard to spiritual care depicts a strong notion that spiritual care was not sufficiently catered for in the Bachelor of Nursing programme. They reported that educators do not worry about how students were going to care for patients in terms of holistic care, rather than spiritual care. Students affirmed that, in their view, the practice of nursing has avoided, or nurses have avoided, the spiritual part of caring when interacting with patients.

Despite students having not learned much about how to deal with a patient’s spiritual needs, they said that in clinical placements it was sometimes inevitable for them to ignore patients when presented with needs that required spiritual intervention. In such situations, they would be pushed to a point where they had to do something. They gave a scenario to prove their point and a second-year student said:

‘I did not learn how to care or comfort people, but in my spirit I knew how to handle the patient’s spiritual needs. I would try to tell the patient not to cry …’ (Female student, 48 years old, 2nd year)

Students’ responses show that they do not know the difference between spiritual and emotional needs. In an effort to clarify the challenge that they are required to render spiritual care, a first-year student expressed the reality of the challenge by stating:

‘… [*H*]ow can I, for example, a 22-year-old male, teach a 44-year-old-woman that what she believes or what she thinks is wrong … because I know that if you have a broken spirit you need to be taught …, how am I going to take the patient from point A to point B in the healing process …’ (Male student, 22 years old, 1st year)

Students felt that spiritual care was more necessary when caring for a dying patient because their presence with the patient and the family was far more important than provision of technical nursing skills and procedures. Students also verbalised concern for being inadequately trained on this caring aspect of nursing which they expressed as a challenge because nursing is a caring profession and yet they learn little about caring. This notion is supported by their perception that they were not well prepared on spiritual matters but rather only sensitised. They felt that the programme was mostly focused on nursing skills competence rather than how to care for the patients holistically and thus were not exposed to spiritual care. Students agreed that they are willing to render spiritual care and acknowledged existing limitations that they face with regard to provision thereof.

Students were asked about how they would want the spiritual care subject to be taught. They were not sure what method they would prefer, although they suggested that a spiritual care subject should be provided beyond first-year level and should be part of the entire curriculum, because they felt there was much more to learn about spiritual care needs. They welcomed the subject of spiritual care, saying that they need to know it because it is important. One student said:

‘… [*P*]atients are in need of spiritual care.’ (Female student, 23 years old, 3rd year)

Another student said:

‘It’s not about me and my problems, I don’t like talking about patient’s problems no, no, but they are in need of it.’ (Female student, 23 years old, 3rd year)

Furthermore, students argued that nurses in clinical practice often are confused about the nature of spiritual care that has to be offered (Narayanasamy & Owens 2001). This confusion is compounded by their lack of understanding of the essence of spiritual care which in turn influences how they deliver it. Students refrained from talking about them and focused on clinical nursing staff because they felt the staff usually hinders them when they are trying to get close to the patients. The students’ assumption was that the staff also need to be educated on spiritual care. This is what they said:

‘We [*nurses*] need to make sure that the nursing staff [*also*] understands that spiritual care is part of the healing process or of health of the patient that it’s not just physical, it’s not just emotional, and [*but*] it’s also spiritual included [*sic*].’ (Female student, 24 years old, 1st year)

A student reported that the clinical staff distract them from trying to build relationships with the patients by assigning them to other tasks. This has a negative or counter-effect on their nursing interventions. They did not welcome the gesture because in their view, it prevented them from practising what they had learned in interpersonal skills learning sessions about the need to build relationships with their patients. Students said that sisters in the wards do not understand spiritual care:

‘The sisters do not want to see you having some time with the patients; when they see you talking with the patients, they will always call you.’ (Female student, 33 years old, 4th year)

‘They just demotivate you … and you don’t know what to do.’ (Female student, 25 years old, 4th year)

These were some of the hindrances that were experienced by students which prevented them from practising spiritual care in clinical placements. Communication is the most vital way to build a relationship with a patient. Therefore, students’ perception that they were being disrupted when they wanted to talk with patients, was affirmation of lack of appropriate understanding about nurse-patient relationships as a basic requirement for spiritual care or the essence of caring.

### Perceived challenges in learning of spiritual care

Regarding the question as to how they would expect spiritual care to be taught, the students provided a wide range of possible teaching-learning processes, including acting out scenarios. Students felt that simulation laboratory (SimLab) was unsuitable for the purpose of acquiring competence in spiritual care. Understandably, students linked spiritual care primarily with faith, religion and God and hence they could not practically apply learning of ‘spiritual care concept’ in the skills laboratory-based learning.

In response to why they regarded simulation as not being ideal, they argued against lack of real feelings in the simulated learning setting. However, they acknowledged that simulation helped them with regard to learning communication skills. Students also advocated that accompaniment by the supervisors could be done in weekly intervals, in addition to regular visits by the supervisors to see how students are progressing in spiritual care matters in clinical practice. They needed guarantees in spiritual care clinical learning as they already had experiences where the professional nurses in the wards interfered in a negative manner when they engaged with patients.

Students reported that few nursing modules deal with the essence of caring, let alone the subject of spiritual care which, according to them, was neither attended to nor addressed when teaching-learning of nursing. Students expressed this opinion by saying:

‘But they [*nurse educators*] don’t teach us how to care yet our profession is a caring profession.’ (Female student, 25 years old, 4th year)

‘They only teach us nursing skills.’ (Female student, 25 years old, 4th year)

The students’ perceptions were that educators do not really pay attention to the need for caring competence and how students would provide holistic nursing in their future practice. This argument was based on the students’ view that holistic or complete caring for patients was lacking in contrast to nursing skills, which received more attention and focus in teaching and learning. Apart from challenges related to classroom learning, junior nursing students felt that it was not easy for them to embark on spiritual matters in their clinical placements because they lacked confidence. However, the demand for religious and spiritual care in the clinical placement is the reality that they deal with every time. A first-year student said:

‘I allowed the patient to go for the prayer; fortunately the prayer time did not clash with the nursing routines because they pray at 13h00 …; because there are no routine procedures that are conducted at that time.’ (Female student, 22 years old, 1st year)

Students also reported that clinical learning of spirituality differed from theoretical learning with regard to the subject of death and dying. They argued that clinical learning experience was completely different from a mental picture they had on the subject of death and dying when it was taught in class. The difference between the realities of theoretical classroom learning and clinical practical learning presented them with a real challenge. The subject of a patient’s death was, in essence, different from book-based knowledge, as it confronted them with a different dimension of understanding. This is what a student said:

‘When I experienced my first [*patient*] death …, you see all the signs that you learn in class patient becomes bluish grey, gasp for air but … I was just shocked … I did not know what was happening until the nurse said get a sheet to wrap the body … I did not know what to do; I was just shocked.’ (Female student, 25 years old, 4th year)

The student reported that it really felt like she had lost a family member and was surprised after that death to realise that she had become so close to the patient. She expressed that she had experienced this in placements whilst she was doing her first year of the nursing progr.:

‘I realised that I did not know much about what I was supposed to do there [*meaning in the death scene*] … I was not fine for some time but I did not know who to turn to in the hospital and on campus.’ (Female student, 26 years old, 4th year)

In response to death-related issues, the student expressed the need to be supported in cases where such incidents occur. The incident of a student who was shocked following observing a patient’s death is an example. According to her, it appeared as though it was not much of an issue to other nursing staff who were present when the patient died. The student said:

‘The nurses just continued work as normal … One nurse pulled me aside and said it does feel like you lost your family member.’ (Female student, 25 years old, 4th year)

Learning about spiritual care or caring from clinical settings, was the most challenging experience. Students proposed that the school of nursing should provide them with support because clinical experience can be too demanding, to a point where they can barely manage.

Another challenge was the clinical environment that was felt to be disruptive, rather than conducive to learning. In fact, they presented the clinical environment as being toxic and demotivating for learning. The students’ perception on this matter was that as time goes on, qualified practice nurses become desensitised to patients’ needs and their behaviour is demotivating to students who are just entering the profession. This is how they perceived the nurses’ behaviour:

‘Disillusion registered nurses that are in the profession already they just make the process worse [*sic*].’ (Female student, 23 years old, 2nd year)

### Perceived conflicting nursing perspectives

The students argued strongly about existing challenges. Despite them being willing to give spiritual care to patients, there are many hindrances that they experience in clinical placements. Different year levels had unique experiences: for example, a final-year student reported perceived professional boundaries as the main hindrance whilst first-year students reported interference by nursing staff as an impediment to their practice of spiritual care. Final-year students reported that they are unable to demonstrate empathy and sympathy to the patient in the way they feel it should be done. This is a result of professionalism that requires students to display empathy, but not sympathy, for patients. They expressed this view, saying:

‘It makes it difficult to truly comfort that patient as to how you feel at that particular moment.’ (Male student, 34 years old, 4th year)

‘We can give sympathy but we have to keep that professional side, so it’s like hugging but also keeping that distance.’ (Male student, 34 years old, 4th year)

‘When it comes to empathy, for instance, it was like in a professional manner; how much can I show towards the patient but it demanded that I must stay professional.’ (Male student nurse, Age 34 years old, 4th year)

Another challenge that students reported, was conflicting principles between ‘spiritual values’ and ‘professional values’. They said that teaching of professional practice informs that they can form friendly relationships with a patient but must not become too close. This is what they said:

‘When you are comforting the patient it is like you are hugging but keeping a distance at the same time.’ (Female student, 27 years old, 4th year)

Showing sympathy and empathy to the patient was fairly acceptable to the students. However, they appealed to be taught how to balance ‘spiritual values’ and ‘professional values’, because usually more focus is placed on the professionalism than caring. Although students acknowledged professionalism as a vital component in nursing, they felt that at times it prohibits genuine interaction with patients. On the contrary to this connotation professional values as a challenge to render spiritual care, students reported a situation where a female patient may feel harassed by a male nurse; yet, in reality the male nurse is trying to comfort the patient. These were some of the inconsistencies that students reported, which they viewed as interfering with spontaneous caring. The final-year students brought insight into a challenge of integration of spiritual care and professionalism or being professional. They said that nursing care is an ongoing process, so they would not think of it as something that should be taught primarily at first-year level but in all year levels. One of the students said:

‘… [*F*]or me, being spiritual is beautiful things, showing compassion and I’ll always do most of those things, but being professional is for me like this rigid type of person. You must learn to communicate … my norms and values …’ (Male student, 35 years old, 4th year)

Students persistently made reference to an existing clash between spiritual care and professional caring principles:

‘… [*L*]ike there is a clash between spiritual care and professional practice. They even tell you must keep a distance between you and the patients.’ (Male student, 33 years old, 4th year)

Students said that these things affected them because they clashed with their personal norms and values.

Other forms of conflict or clashes that students experienced included required participation in nursing procedures that were not in line with their own belief system. Expected participation in termination of pregnancy and administration of blood procedures as nursing duties were some of the most contradictory scenarios. However, they said that they would find ways to adapt to those situations:

‘You must forget about yourself at that point and give care that the patient requires because at point in time there’s nobody else who can do that for the patient so you have to think for the patient.’ (Female student, 23 years old, 4th year)

They attested about situations where they had to set aside their own feelings and attend to patients’ needs. They felt compelled to participate as duty called. Discrepancies between professional practice requirements and willingness to offer spiritual care were also reflected in the literature. The students expressed the need to find ways in which they could best deal with such clinical situations. They said:

‘We need to find our balance.’ (Male student, 35 years old, 4th year)

This expression was based on their acceptance that they needed to offer required care in spite of contradiction of their own beliefs, norms and values. This gap was reported by Puchalski (2001), who attested that despite nurses not being prepared to provide spiritual care, they are called on daily to respond to the spiritual needs of their patients. The students verbalised that it is difficult for them to provide emotional support or spiritual care to patients because of religious and cultural diversity. The students were concerned that they might misdirect the patient in his future life, especially in the case of a dying patient. They realised that they are actually not taught how to care for a suffering person, but only to provide technical nursing. This is what a student said in this respect:

‘They [*nurse educators*] teach you [*student*] technical things to make you a professional but they don’t teach you about caring because our programme is BCUR – Latin for Baccalaureus Curationis. It’s a degree in caring, but I don’t think they teach you how to care.’ (Female student, 26 years old, 4th year)

### Students’ spiritual and emotional needs

Students made it clear that when a sick person knows that someone cares and regards them as important, it connects to their psychological and social wellbeing. They feel boosted that they could care for the person holistically. According to students, spiritual care is not only important to patients but to them as well. One student reported a situation to demonstrate how she as a nurse feels when caring for a patient in a manner that is meaningful to her:

‘When I talk to my patients and they say to me thank you or when they appreciate me for services [*rendered*] it makes me feel good and I’m looking forward to go back there tomorrow to offer what I have.’ (Female student, 22 years old, 1st year)

The students expressed that the moments they spend with the patient are rewarding because patients appreciate being helped. This response gives the students a good feeling, knowing that they have helped somebody. The students acknowledged that when they are appreciated by the patients it gives them satisfaction; also knowing that the patients need them to be there gives students satisfaction (Burnard 1988).

Students indicated that when they came into the nursing profession they were all excited with an elevated sense of spiritual enlightenment and desire to help patients. However, there are situations that happen in the clinical placements that break students down. Eventually, they cannot keep up with their spiritual commitment to care, but just feel like doing whatever that is being done in the clinical practice. They depicted a need to be helped in this matter. This is what they said:

‘So we need to be strengthened here within our learning institution.’ (Female student, 26 years old, 4th year)

Contrary to Burnard (1988), who argued that nurses should clarify their own spiritual beliefs or lack of beliefs in order to help clients to engage spiritually, the participants in the study pointed at a need for *them* to be cared for as well, in order for them to care for someone *else*. They expressed the need for emotional support from the clinical supervisors when they are in the clinical placements. They said the following:

‘… [*E*]ven if it means the supervisor spends just thirty minute session sitting with the students as a group listening to issues, scenarios and questions that students have. The supervisor may give advice and probably bring up a suggestion on what a student would need to do in a situation like this and that.’ (Female student, 26 years old, 4th year)

Student nurses expressed the need to be cared for spiritually. They did not only relate to personal challenges that they come with but also with the need to meet nursing demands and expectations which were experienced as enormous, especially when they deal with patient values that supersede their personal values and which require them to comply with what is expected behavi*o*ur. They said:

‘We just push our feelings aside and do the job that’s supposed to be done. I think and [*sic*] that’s why nurses become closed and they have breakdowns.’ (Female student, 23 years old, 3rd year)

‘Dealing with personal values and client values and expectations, for example, you don’t believe in abortion, you don’t like abortion, you value life but you find yourself compelled to participate for [*the*] patient’s sake.’ (Female student, 23 years old, 3rd year)

Despite unwelcoming experiences about learning and practice of spiritual care, students expressed the need for more guidance on how to provide spiritual care and professional care values without feelings of mutual exclusiveness. Students were also concerned about their own personal challenges:

‘Sometimes if you [*students*] come with your baggage, like they say if you had a bad day and come to the hospital, you’re not going to treat your patients the same way that you would have wanted to treat them like on a good day because you’re bringing your baggage from your personal life into your professional life.’ (Female student, 33 years old, 4th year)

## Ethical considerations

Certification clearance no. 13/4/22 to conduct the study was obtained from the Community of Health Sciences Faculty and Senate Higher Degree Research Committees from the University of the Western Cape Province. The participants were provided with information sheets and informed about their right to withdraw from participation at any time without any negative effects. Participants voluntarily gave written informed consent and signed binding confidentiality agreements to protect sensitive issues. Participants were further protected from unwarranted physical or mental discomfort, distress, harm, danger, cohesion or deprivation by answering their questions in a manner that promoted mutual trust and respect. Audio-tapes and transcripts were locked in a safe place and would be destroyed five years after publishing the study results, according to the university policy.

## Trustworthiness

### Rigour

Trustworthiness was used to ensure rigorous qualitative research. The four aspects of the model of trustworthiness, namely, truth value, applicability, consistency and neutrality (Klopper 2008), were observed. *Truth value* in this study was established by use of scientific strategies and methods, namely, a generative-explorative-descriptive-contextual design which ensured that participants were able to express their views freely. A thick description of the participant’s experiences about learning of spiritual care in the clinical placements was included. *Applicability* refers to the generalisability of findings according to Creswell (2009:300) and Klopper (2008). Despite the nebulous nature of the concept of spirituality and consequently spiritual care, applicability to similar contexts can be realised. It is believed that the results of this study are transferable to other schools of nursing at higher education institutions, as nursing curricula are – to a certain degree – aligned in South Africa. *Consistency* is acknowledged by the fact that the participants shared lived experiences in the environment familiar to them, which promoted calmness. *Neutrality* was ensured by use of scientific procedures and an audit trail to keep data free from contamination. Reflexivity, bracketing, verification and validation of data during interviews were used to ensure rigour. Furthermore, codes, summaries and themes were validated together by both the researcher and the independent coder.

## Discussion

The findings of the study indicated different views from year levels one to four, with unique experiences. The common view of the entire student population was the shallowness and brevity of the subject matter or content in the undergraduate programme. Final-year students reported perceived professional boundaries as the main hindrance to practising spiritual care, as these boundaries prevented them from showing empathy and sympathy to the patient in the way they felt they should. They associated the hindrance with professionalism that requires them to exercise caution in maintaining an interpersonal distance between themselves and the patient. In their view, this made it difficult to truly comfort the patient in a particular way at that particular moment. On the other hand, first-year students reported interference by nursing staff as being the problem that fractured their implementation of spiritual care. These findings are in keeping with reported challenges that lead to lack of teaching and practice of spiritual care in Nursing (Baldacchino 2006:888; Ross 2006).

Grosvenor (2000:29) argues that spiritual care has always been an integral part of holistic care, which is the very heart of nursing. However, the challenge lies in failure to acknowledge that learning holistic care values is caught rather than taught. In line with Grosvenor’s (2000) view, it is acknowledged that the spiritual dimension of care which is inextricably tied to the ethic of care, is also mostly caught rather than taught. However, the most echoed issue is the loss of spiritual care in nursing, which is assumed to result from nursing models that do not explicitly include spirituality. Misunderstanding amongst nurses was reported as another possible cause for existing inconsistences in teaching and practice of the spiritual aspect of care in the nursing profession. Participants reported that spiritual subject matter was very scarce, brief and shallow and they were not informed about the purpose for providing religious and or spiritual care. Martsolf and Mickley (1998:294) reported the similar occurrence where nurses are confused in the teaching and practice of spiritual care because of a lack of clarity on the concept of spiritual care. Other reported causes for lack of practice of spiritual care include social challenges, where individuals are not accustomed to expressing their faith. Professionalism, increasing pressure as a result of high patient turn over, increasing use of technology and bureaucratic paperwork were also reported as compounding the situation of compromised spiritual care (Grosvenor 2000:31).

Despite reported experiences of challenges and inconsistences in practice of spiritual care, the participants reported that they were pushed to engage with patients’ spiritual matters, despite not being formally prepared for spiritual care. In view of students’ reported experience of being pushed to participate in the patients’ spiritual matters, an assumption is made that reality of the circumstance at the time would not allow student to refuse to offer required spiritual care. This occurrence supports Van Leeuwen and Cusveller’s (2003:235) argument that nurses can no longer be stuck with lack of a universal definition of spiritual before they could consider to offer it because the patient are already in desperate need of it. The authors express the notion that the need for a definition of spiritual care, which could delay its implementation based on perceived differences, can be bypassed by adopting a functional definition of spirituality. A functional spirituality simply refers to what works out at a given time and which would be acceptable to both the nurse and patient, as opposed to a definition of spiritual care with a fixed meaning and notions of spiritual care; whilst a universal definition is sought and nursing education contemplates the formal spiritual care in the undergraduate nursing programme. Students expressed the need to be cared for spiritually because at times the patients’ needs require care that is beyond their means. This means that the demand from clinical settings sometimes supersedes what they can offer. Students come to the profession with their own burdens. Van Leeuwen and Cusveller (2003:236) suggest that nurses’ spirituality should be taken into account in the nursing profession, where they should be given space for their own feelings and convictions which they might have when caring for patients’ spirituality.

Regarding teaching and learning of spiritual care, the participants suggested a number of ways by which the spiritual care subject matter could be taught and learned. They suggested using case-based education where cases and scenarios could guide students in embarking on problem-solving exercises to deal with patient spiritual matters, including medical dilemmas. Discussion in classroom and clinical settings can be encouraged when the nurse educators accompany students with an intention to take the student through relevant spiritual learning opportunities in the clinical facilities as a priority and not only focusing on assessing them on clinical nursing skills. Despite caring being perceived as important for the practice of nursing, students’ experiences indicated that learning ‘nursing skills’ and ‘nursing procedures’ outweighed learning of ‘how to care’, which in their view should be an integral part of nursing practice. This suggestion would mean that nurses are prepared and understand the essence of nursing as opposed to purely technical skills nursing. Van Leeuwen and Cusveller (2003:237) provide a process through which spiritual care can be taught and provided. Based on the competencies for spiritual care, Van Leeuwen and Cusveller (2003:237) present a competency profile which depicts three domains relating to the personhood of the nurse. They include the nurse’s attitude and personal qualities, professional responsibility and knowledge. In line with the nursing process, nurses can collect information about a patient’s spirituality in order to identify spiritual needs and plan accordingly. A nurse’s conviction and non-judgemental attitude are of primary importance in maintenance of a conducive environment for spiritual care (Van Leeuwen et al. 2006).

## Limitations

### Lack of clarity on spiritual subject matter

A serious limitation was the fact that some students could not comprehend the subject matter of spirituality outside religious confinements; which possibly compromised any rich discussion topics that otherwise would have been covered. This pointed to an oversight with regard to covering such an import aspect of nursing. This lack could be assumed to be associated with the fact that spiritual care is not formally taught in the undergraduate nursing programme in the Western Cape and South Africa in general.

#### Suggestion for future research

More research needs to be done in this area as a means to sound a siren about a possibly forgotten aspect of nursing practice.

### Need for students to reflect on spiritual subject matter and discussions

Some students wanted more discussion sessions. They needed individualised reflections after group discussion as their awareness level about spiritual care in nursing practice was heightened to the point that some not only experienced guilt feelings and blaming but also wanted to know how soon and how best the void [absence of spiritual care] in nursing could be filled and the gap be bridged. In retrospect, this was a limitation because the researcher did not make provision for ongoing sessions, debriefing sessions or continuous FG discussions – the students appeared to have enjoyed the ones they experienced because they asked for subsequent meeting dates in order to continue their discussion. This was not possible because the FG discussion was only designed to collect data for the study and nothing else. In future research, the researcher may create on-line discussion groups to allow and assists student to continue to engage with the subject matter. As a matter of fact, this method can be used to socialise students with regard to spiritual care being seen as an aspect of holistic nursing, which is the cornerstone of the nursing profession.

## Recommendations

It is, therefore, recommended that nurse practitioners should strive to improve nursing practice through application of spiritual care, which is currently a missing component of holistic nursing. It is believed that spiritual care will complete the essence of caring in the nursing profession. Nurses are urged to engage more on the subject and conduct research that needs to be done on spiritual care in nursing, as it is still an undeveloped area in healthcare in South Africa.

## Conclusion

Participants attested to the subject of death and dying and bereaved families as the most explored area, even though it was done over only one semester of a four-year period during the programme. Their point of departure was to relate how spiritual care applies to their own lives and how they would implement it when practising nursing. They were not clear as to how spiritual care as a subject could be taught and learned. However, they expressed a need to learn more about it because the need for spiritual care in health facilities is currently not being met.
